# Loss of fatty acid binding protein 3 ameliorates lipopolysaccharide-induced inflammation and endothelial dysfunction

**DOI:** 10.1016/j.jbc.2023.102921

**Published:** 2023-01-19

**Authors:** Hien C. Nguyen, Shuhan Bu, Sepideh Nikfarjam, Berk Rasheed, David C.R. Michels, Aman Singh, Shweta Singh, Caroline Marszal, John J. McGuire, Qingping Feng, Jefferson C. Frisbee, Mohammad Qadura, Krishna K. Singh

**Affiliations:** 1 Department of Medical Biophysics, Schulich School of Medicine & Dentistry, Western University, London, Ontario, Canada; 2 Department of Anatomy and Cell Biology, Schulich School of Medicine & Dentistry, Western University, London, Ontario, Canada; 3 Department of Applied Science, Fanshawe College, London, Ontario, Canada; 4 Physiology and Pharmacology, Schulich School of Medicine & Dentistry, Western University, London, Ontario, Canada; 5 Institute of Medical Science, University of Toronto, Toronto, Ontario, Canada

**Keywords:** endothelial cell, fatty acid binding protein 3, lipopolysaccharide, endothelial dysfunction, AKT, protein kinase B, CVD, cardiovascular diseases, eNOS, endothelial nitric oxide synthase, FABPs, fatty acid–binding proteins, HUVECs, human umbilical vein endothelial cells, LPS, lipopolysaccharide, NO, nitric oxide, PAD, peripheral artery disease, PPAR, peroxisome proliferator-activated receptor, qPCR, quantitative polymerase chain reaction, rhFABP3, recombinant human FABP3

## Abstract

Circulating fatty acid–binding protein 3 (FABP3) is an effective biomarker of myocardial injury and peripheral artery disease (PAD). The endothelium, which forms the inner most layer of every blood vessel, is exposed to higher levels of FABP3 in PAD or following myocardial injury, but the pathophysiological role of endothelial FABP3, the effect of FABP3 exposure on endothelial cells, and related mechanisms are unknown. Here, we aimed to evaluate the pathophysiological role of endothelial FABP3 and related mechanisms *in vitro*. Our molecular and functional *in vitro* analyses show that (1) FABP3 is basally expressed in endothelial cells; (2) inflammatory stress in the form of lipopolysaccharide (LPS) upregulated endothelial FABP3 expression; (3) loss of endogenous FABP3 protected endothelial cells against LPS-induced endothelial dysfunction; however, exogenous FABP3 exposure exacerbated LPS-induced inflammation; (4) loss of endogenous FABP3 protected against LPS-induced endothelial dysfunction by promoting cell survival and anti-inflammatory and pro-angiogenic signaling pathways. Together, these findings suggest that gain-of endothelial FABP3 exacerbates, whereas loss-of endothelial FABP3 inhibits LPS-induced endothelial dysfunction by promoting cell survival and anti-inflammatory and pro-angiogenic signaling. We propose that an increased circulating FABP3 in myocardial injury or PAD patients may be detrimental to endothelial function, and therefore, therapies aimed at inhibiting FABP3 may improve endothelial function in diseased states.

The fatty acid–binding proteins (FABPs) are a family of transport proteins for fatty acids and other lipophilic substances between extracellular and intracellular membranes and receptors and play an important role in the regulation of lipid homeostasis (1). FABPs are also involved in the production of the cell membrane in the endoplasmic reticulum and various enzymatic activities in the cytosol (2). The FABP protein superfamily is encoded by nine different genes, and different FABPs have usually been named according to their dominant expression in certain tissues (3), of which FABP4 and FABP5 are reported to be expressed in the endothelial cells (1, 4), where they play overlapping and nonredundant roles. They are pro-angiogenic proteins and modulate important signaling pathways, including p38, eNOS, and peroxisome proliferator-activated receptor (PPAR) δ signaling (1, 4).

The myocardial isoform, heart-type fatty acid–binding protein, is encoded by the *FABP3* gene. Besides its abundant expression in the cardiomyocytes, FABP3 is also expressed significantly in other cell types (5). Their lipid-trafficking mechanism is essential for the metabolic homeostasis of cardiac function (6). For their unique cardiac-expression profile, FABP3 has been proposed as an effective biomarker of myocardial injury (7) as FABP3 is readily released from heart muscles into the blood following a heart attack (8, 9, 10). The release of FABP3 from the injured myocardium has been observed in both animal models (11) and myocardial infarction patients (12). Aside from the general lipid-trafficking mechanism and its feature as a cardiac biomarker, the unique function of FABP3 remains largely unknown, particularly its roles in cardiovascular diseases (CVDs). Systemic infections, or sepsis, have been reported to exacerbate cardiac injuries in atherosclerotic patients (13). Physiologically, the body’s lipids contribute not only as an efficient source of energy but also as a source of regulatory signals maintaining proper systemic functions or homeostasis, such as hormonal balance (14) and inflammation (15). Pathologically, lipids bioavailability and their interacting factors are the driving agents of the metabolic syndrome (16). Moreover, the bioavailability of lipids and their interacting factors have been employed as biomarkers for cardiovascular-related complications (17).

Accordingly, recently we identified increased circulating levels of FABP3 in peripheral arterial disease (PAD) patients with severe inflammation and particularly undergoing critical limb ischemia, who were negative for any signs of cardiac damage (18). The endothelium lines the inner walls of all blood vessels and is in direct contact with blood and regulates tissue–blood metabolic and signaling exchanges, vascular homeostasis, and inflammation; impaired endothelial function or endothelial dysfunction is a key mechanism behind CVDs (14, 19, 20). It is important to note that in both myocardial ischemia and PAD patients, endothelial cells are directly exposed to higher levels of FABP3 (8, 9, 10). However, the source of FABP3 and its effect on the endothelium remains largely unknown, and the role of endothelial FABP3 has not been fully characterized at baseline and under stress conditions. Accordingly, our objective is to evaluate (1) the endothelium as a potential source of FABP3, (2) the role of endothelial FABP3 in endothelial function and survival, and (3) the effect of increased FABP3 exposure on endothelial cell function and inflammation at baseline and after stress and (4) related mechanisms.

Chronic inflammation is the central driving mechanism between endothelial dysfunction and CVDs (21, 22). Inflammation is also a common factor between myocardial ischemia/heart failure (23) and PADs (24), which are associated with increased circulatory FABP3 and thereby increased FABP3 exposure to endothelial cells. Lipopolysaccharide (LPS), a Gram-negative bacterial endotoxin, is known to induce severe inflammation and endothelial dysfunction (25); accordingly, LPS is extensively used in experimental models to study inflammation and associated endothelial dysfunction *in vitro* and *in vivo* (25, 26, 27, 28).

Our data demonstrate that endothelial cells basally express FABP3; inflammation, in the form of LPS treatment, significantly upregulates endothelial FABP3 expression. Furthermore, loss-of-endothelial FABP3 inhibits LPS-induced endothelial dysfunction by promoting cell survival and anti-inflammatory and pro-angiogenic pathways. In contrast, gain-of-endothelial FABP3 appears to exacerbate inflammation and endothelial function. Our results suggest that elevated FABP3 in myocardial injury or PAD may be detrimental to the endothelium; therefore, therapies aimed at inhibiting serum FABP3 may improve endothelial function in diseased states.

## Results

### LPS upregulates FABP3 expression in endothelial cells

**Figure 1:**
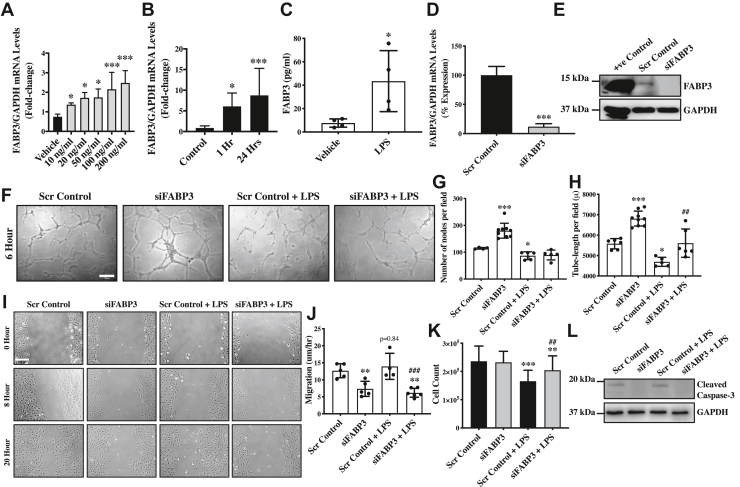
**LPS-induced FABP3 modulates endothelial function**. *A*, HUVECs were treated with different doses of LPS, and RNA was extracted 24 h posttreatment to perform qPCR for *FABP3*. *B*, HUVECs were treated with 100 ng/ml of LPS, and RNA was extracted 1h and 24 h post-treatment to perform qPCR for *FABP3*. *C*, HUVECs were treated with 100 ng/ml of LPS for 24 h, and culture media were collected to perform ELISA for *FABP3*. *D* and *E*, HUVECs were transfected with either scrambled control or siFABP3, and RNAs and proteins were extracted to perform qPCR and immunoblot, respectively, for FABP3; GAPDH was used as a control. *F*–*H*, HUVECs were transfected with either scrambled control or siFABP3 and seeded on Matrigel in the presence of vehicle or LPS for 6 h, and tube formation was assessed microscopically (F); the number of nodes (*G*) and tube lengths (*H*) were quantified (scale bar = 100 μm). *I* and *J*, HUVECs were transfected with either scrambled control or siFABP3, and 24 h posttransfection, a scratch was made, and cell migration was assessed using phase contrast light microscopy at 0, 8, and 20 h, scale bar = 200 μm (*I*), and migratory capacity was calculated (*J*). *K*, HUVECs were transfected with either scrambled control or siFABP3 for 24 h, and the live cells were counted using Cytosmart automated cell counter. *L*, HUVECs were transfected with either scrambled control or siFABP3, and then proteins were extracted to perform immunoblot for cleaved-CASPASE3 and GAPDH (loading control). Difference between the means of two groups and more than two groups were calculated using the Student’s *t* test and one-way ANOVA with Tukey’s multiple comparison test, respectively. ∗*p* < 0.05, ∗∗*p* < 0.01, ∗∗∗*p* < 0.001 *versus* Vehicle, control and Scr Control. #*p* < 0.05, ##*p* < 0.01, ###*p* < 0.001 *versus* Scr Control + LPS. N = 3 in triplicates for qPCR. Data are represented as mean ± SD. FABP3, fatty acid–binding protein 3; HUVECs, human umbilical vein endothelial cells; LPS, lipopolysaccharide; qPCR, quantitative polymerase chain reaction.

Our *FABP3* quantitative polymerase chain reaction (qPCR) data on vehicle-treated (control) endothelial cells confirmed the basal expression of *FABP3* in human umbilical vein endothelial cells (HUVECs) (Fig. 1*A*). Next, to evaluate the effect of inflammation in endothelial cells in the form of LPS-treatment on FABP3 expression, we treated endothelial cells with different doses of LPS (10, 20, 50, 100, and 200 ng/ml) or vehicle control for 24 h and then measured the *FABP3* expression. Our qPCR data show significant upregulation of *FABP3* in endothelial cells by all the doses of LPS-treatment (Fig. 1*A*). Maximum but similar *FABP3* expression was observed for 100 and 200 ng/ml of LPS, and accordingly, 100 ng/ml was chosen to be the experimental dose to evaluate the effect of loss of FABP3 on LPS-induced endothelial dysfunction. A similar dose has been used by many other comparable studies in endothelial cells (29, 30). We also evaluated the effect of time on LPS-induced *FABP3* upregulation and observed that the *FABP3* was upregulated as early as 1-h posttreatment (Fig. 1*B*). We then tested whether LPS-induced *FABP3* upregulation is associated with increased secretion of FABP3 in the culture medium and observed increased LPS treatment–induced secretion of FABP3 in the culture medium (Fig. 1*C*).

### Endothelial cell-specific loss of FABP3 protects against LPS-induced endothelial dysfunction and apoptosis

To understand the effect of LPS-induced upregulation of FABP3 on endothelial function, we successfully silenced *FABP3* in HUVECs and observed ∼90% reduction at the transcript level (Fig. 1*D*). FABP3-silencing was also confirmed at the protein level by Western blotting for FABP3 (Fig. 1*E*). We then treated FABP3-silenced and control endothelial cells with 100 ng/ml of LPS and evaluated endothelial function in the form of tube-forming, migratory, and proliferative potential of endothelial cells. To our surprise, the loss of FABP3 significantly increased the number of nodes and tube length in FABP3-silenced *versus* control endothelial cells (Fig. 1, *F*–*H*). LPS treatment is known to inhibit tube-forming potential (31); accordingly, we also observed significant inhibition of tube formation in LPS-treated scrambled-transfected *versus* vehicle-treated scrambled-transfected control endothelial cells (Fig. 1, *F*–*H*). Interestingly, loss of FABP3 was able to significantly restore tube length in LPS-treated FABP3-deficient in comparison to LPS-treated control endothelial cells (Fig. 1, *F* and *H*). However, the loss of FABP3 showed no effect on the LPS-induced inhibition of the number of nodes in HUVECs (Fig. 1, *F* and *G*). Next, to understand the effect of LPS treatment on the migratory capacity of FABP3-deficient endothelial cells, we measured migratory capacity *via* scratch assay (32). Loss of FABP3 and LPS treatment appeared to inhibit and upregulate endothelial cell migration, respectively (Fig. 1, *I* and *J*). LPS-induced upregulation of endothelial cell migration has been previously reported depending on specific dosages (33); however, loss of FABP3 was able to attenuate LPS’s effect on endothelial cell migration (Fig. 1, *I* and *J*). We did observe a trend toward increased LPS-induced migration, but the difference was nonsignificant and that can be attributed to the sensitivity of the method used. We then evaluated the effect of loss of FABP3 and LPS on the proliferative capacity of endothelial cells *via* measuring the cell count using the CytoSmart Automated Cell Counter. Loss of FABP3 appeared not to affect endothelial cell proliferation; however, LPS treatment significantly inhibited the proliferative potential of endothelial cells, which was, interestingly, restored in the FABP3 silenced and LPS treated in comparison to LPS-treated scrambled control-transfected endothelial cells (Fig. 1*K*). Next, to understand whether LPS-induced reduced cell proliferation is associated with increased cell death and whether the loss of FABP3 is associated with the restoration of cell proliferation is due to increased survival, we measured apoptosis in FABP3-silenced and LPS-treated endothelial cells. Our Western blot data demonstrated the absence of cleaved-CASPASE3 protein in the siFABP3-transfected endothelial cells, suggesting that LPS-induced apoptosis in endothelial cells was inhibited by loss of FABP3 in LPS-treated endothelial cells (Fig. 1*L*). Overall, these data indicate that loss of FABP3 protects against LPS-induced endothelial dysfunction by restoring angiogenic, migratory, and proliferative potential and by inhibiting LPS-induced apoptosis of endothelial cells.

### Endothelial cell-specific loss of FABP3 restores LPS-induced endothelial nitric oxide synthase expression and activation

**Figure 2:**
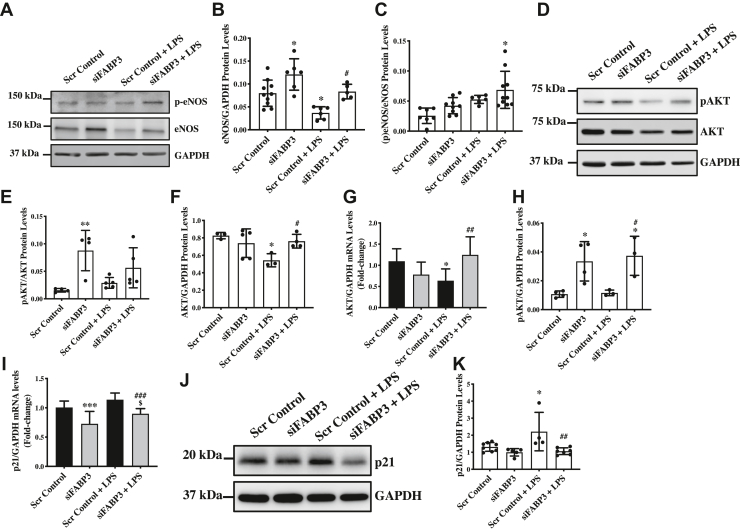
**Endothelial cell-specific loss of FABP3 promotes eNOS expression and activation**. HUVECs were transfected with either scrambled control or siFABP3 for 24 h and treated for additional 24 h with LPS, and then protein and RNA were extracted to perform immunoblot and qPCR, respectively. *A*–*C*, immunoblotting for eNOS, p-eNOS, and GAPDH (*A*), and quantification for eNOS (*B*) and p-eNOS/eNOS ratio (*C*). *D*, *E*, *F* and *H*, immunoblotting for AKT, pAKT, and GAPDH and quantification for pAKT/AKT ratio (*E*), AKT (*F*), and pAKT (*H*). *J* and *K*, immunoblot (*J*) and (*K*) quantification for p21. *G* and *I*, qPCR was performed for *AKT* (*G*) and *p21* (*I*). Difference between the means of groups were calculated using one-way ANOVA with Tukey’s multiple comparison test. ∗*p* < 0.05, ∗∗*p* < 0.01, ∗∗∗*p* < 0.001 *versus* Scr Control. #*p* < 0.05, ##*p* < 0.01, ###<p0.001 *versus* Scr Control+LPS. $*p* < 0.05 *versus* siFABP3. N = 3 in triplicates for qPCR, and data are represented as mean ± SD. AKT, protein kinase B; eNOS, endothelial nitric oxide synthase; FABP3, fatty acid–binding protein 3; HUVECs, human umbilical vein endothelial cells; LPS, lipopolysaccharide.

To understand the effect of loss of FABP3 and LPS on the molecular and regulatory level in endothelial cells, we evaluated the expression and activation of the essential regulators of endothelial function. Endothelial nitric oxide synthase (eNOS) and protein kinase B (AKT) are the two essential regulators of endothelial function (34). LPS is known to inhibit eNOS expression and activation (35), and accordingly, we also observed a reduction in the eNOS protein expression and activation levels in LPS-treated endothelial cells (Fig. 2, *A*–*C*). Interestingly, we observed a significantly higher protein level of eNOS in FABP3-silenced endothelial cells, which also corresponded with increased phosphorylation of eNOS (Fig. 2, *A*–*C*). LPS-associated inhibition of eNOS expression and activation was restored in LPS-treated FABP3-silenced endothelial cells (Fig. 2, *A*–*C*). Given that the phosphatidylinositol 3-kinase (PI3K)/AKT/eNOS signaling pathway is critical for the maintenance of endothelial function and that activated AKT can directly activate eNOS (34), we next measured total and activated AKT levels in FABP3-silenced and LPS-treated endothelial cells. LPS has been shown to compromise AKT activation (36); accordingly, we also observed reduced AKT activation in LPS-treated endothelial cells (Fig. 2*D*). However, to our surprise, when we quantified and evaluated the activated *versus* total AKT, the inhibition was not significant between the LPS-treated siFABP3- and scrambles-transfected HUVECs (Fig. 2*E*). Next, we questioned whether this lack of difference is due to inhibition of total AKT expression by LPS treatment in endothelial cells and quantified total AKT. As expected, LPS significantly inhibited total AKT expression in endothelial cells (Fig. 2*F*). Interestingly, AKT expression was restored in LPS-treated FABP3-silenced endothelial cells (Fig. 2*F*), and when we quantified activated AKT (p-AKT), we observed a significant upregulation again for both FABP3-silenced endothelial cells and LPS-treated FABP3-silenced endothelial cells (Fig. 2, *D* and *H*). Protein p21, a cell cycle inhibitor, is known to regulate endothelial cell proliferation physiologically and also in pathological conditions (37). Most importantly, LPS-mediated inhibition of cell proliferation has been previously attributed to p21 upregulation (38). Accordingly, we measured the p21 expression in FABP3-silenced and LPS-treated endothelial cells. Our transcript data showed a significant reduction in *p21* transcript level in FABP3-silenced endothelial cells; p21 transcript and protein appeared to be upregulated in LPS-treated endothelial cells, whereas the p21 expression was restored in LPS-treated FABP3-silenced endothelial cells in comparison to LPS-treated scrambled control-transfected endothelial cells (Fig. 2, *I*–*K*). These data indicated that loss of FABP3-associated restoration of endothelial function in LPS-treated endothelial cells is mediated by increased AKT/eNOS signaling and inhibition of LPS-associated p21 expression.

### Endogenous FABP3 deficiency ameliorates LPS-induced inflammation in endothelial cells

**Figure 3:**
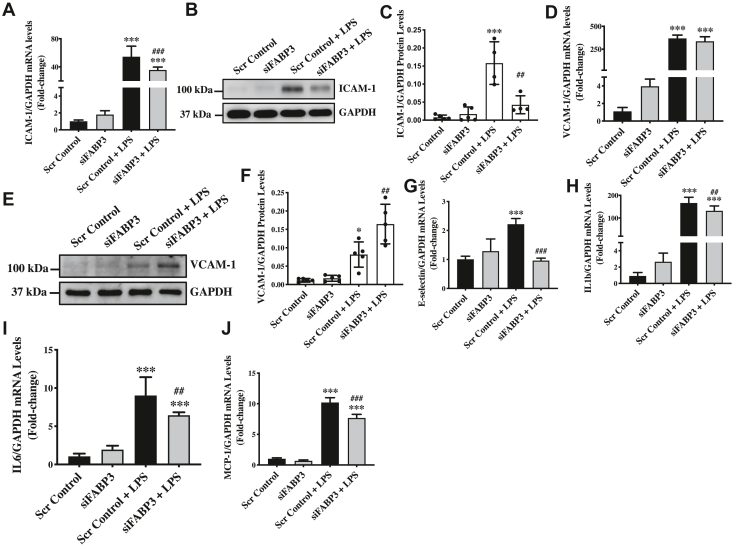
**Inflammatory markers modulated by loss of FABP3's function in LPS-treated endothelial cells**. HUVECs were transfected with either scrambled control or siFABP3 for 24 h and treated for additional 6 h and 24 h with LPS to isolate RNA and protein, respectively. *A*, *D*, *G*–*J*, bar graphs representing qPCR data for *ICAM-1* (*A*), *VCAM-1* (*D*), *E-SELECTIN* (*G*), *IL1b* (*H*), *IL6* (*I*), and *MCP-1* (*J*). *B*, *C*–*F*, immunoblot and quantification for ICAM-1 (*B* and *C*) and ICAM-1 (*E* and *F*). Difference between the means of groups were calculated using one-way ANOVA with Tukey’s multiple comparison test. ∗*p* < 0.05, ∗∗*p* < 0.01, ∗∗∗*p* < 0.001 *versus* Scr Control. ##*p* < 0.01, ###*p* < 0.001 *versus* Scr Control+LPS. N = 3 in triplicates for qPCR, and data are represented as mean ± SD. FABP3, fatty acid–binding protein 3; HUVECs, human umbilical vein endothelial cells; LPS, lipopolysaccharide; qPCR, quantitative polymerase chain reaction.

To assess the role of FABP3 in endothelial inflammation, we evaluated the expression level of key inflammatory markers, including the ICAM-1, VCAM-1, and E-SELECTIN, and the secretory inflammatory cytokines, such as IL1b, IL6, and MCP-1, in FABP3-silenced and LPS-treated endothelial cells. LPS-treatment is known to induce ICAM-1 and VCAM-1 expression (39); accordingly, we also observed a significant induction of ICAM-1 (Fig. 3, *A*–*C*) and VCAM-1 (Fig. 3, *D*–*F*) in the LPS-treated scrambled control-transfected endothelial cells. Loss of FABP3 significantly inhibited LPS-induced expression of ICAM-1 at both the transcript and protein levels in HUVECs (Fig. 3, *A*–*C*). LPS-induced *VCAM-1* transcript level also appeared to be inhibited by loss-of FABP3 in endothelial cells; however, to our surprise, these data did not translate to the protein levels, where we observed further increased level of VCAM-1 in the LPS-treated FABP3-silenced endothelial cells *versus* LPS-treated scrambled control-transfected endothelial cells (Fig. 3, *D*–*F*). Similar to ICAM-1 and VCAM-1, the expression level of E-SELECTIN was induced by LPS, which was again restored by loss of FABP3 in LPS-treated FABP3-silenced endothelial cells (Fig. 3*G*). LPS is also known to promote the expression of inflammatory cytokines, such as interleukins, IL1b and IL6, and the chemoattractant factor MCP-1 (40). Accordingly, we observed LPS-induced significant upregulation in the expression level of *IL1b and IL6* along with the expression of *MCP-1* in endothelial cells (Fig. 3, *H*–*J*). Interestingly, loss of FABP3 was successfully able to significantly inhibit the expression of all these studied inflammatory molecules in LPS-treated FABP3-silenced endothelial cells (Fig. 3, *H*–*J*). Taken together, these data indicate that loss of FABP3 protects against LPS-induced inflammation in endothelial cells.

### Exogenous exposure of FABP3 exacerbates LPS-induced inflammation in endothelial cells

**Figure 4:**
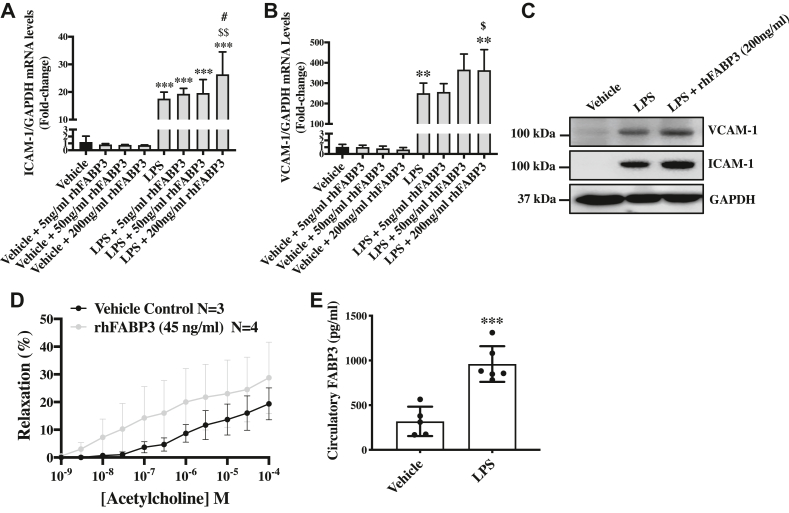
**Exogenous FABP3 treatment exacerbates LPS-induced inflammation in endothelial cells, and LPS treatment upregulates circulatory FABP3 levels in wildtype mice**. HUVECS were cultured, and following 60 to 70% confluency, these cells were pretreated with different doses of recombinant human FABP3 for 1-h before treatment with 100 ng/ml of LPS. Later, 6 h and 24 h posttreatment, RNA and proteins, respectively, were extracted. *A* and *B*, bar graphs show the qPCR quantification for *ICAM-1* (*A*) and *VCAM-1* (*B*). *C*, the qPCR data were further confirmed by immunoblotting for ICAM-1 and VCAM-1, which also showed exacerbation of ICAM-1 and VCAM-1 expression in rhFABP3 pretreated and LPS-treated endothelial cells. *D*, isometric tension data from myograph experiments using acetylcholine to show relaxation (%) of phenylephrine-contracted aorta in control (PBS) *versus* rhFABP3 (45 ng/ml, 20 min) groups (*p* > 0.05). *E*, wildtype mice were treated with vehicle (N = 5) or LPS (N = 6, 4 mg/kg), and plasma was collected 4 h posttreatment to perform ELISA for *FABP3*. Difference between the means of groups were calculated using one-way ANOVA with Tukey’s multiple comparison test (*A* and *B*), two-way ANOVA with Bonferroni’s multiple comparison test (*C*), and Student’s *t* test (*D*). ∗∗*p* < 0.01, ∗∗∗*p* < 0.001 *versus* Vehicle. *$p* < 0.05, $$*p* < 0.01 *versus* LPS + 50 ng/ml rhFABP3, #*p* < 0.005 *versus* LPS + 5 or 50 ng/ml rhFABP3. Data are represented as mean ± SD except in figure *D*, which is represented as mean ± SEM. FABP3, fatty acid–binding protein 3; HUVECs, human umbilical vein endothelial cells; LPS, lipopolysaccharide; rhFABP3, recombinant human FABP3.

Next, to understand the effect of exogenous exposure of FABP3 on endothelial inflammation basally and after LPS-stimulation, we treated endothelial cells with different doses of recombinant human FABP3 (rhFABP3) and LPS and then measured the expression level of ICAM-1 and VCAM-1. Recombinant human FABP3 alone did not significantly affect the inflammation, measured in the form of ICAM-1 and VCAM-1 expression; however, rhFABP3 significantly increased *ICAM-1 and VCAM-1* transcripts in LPS-treated endothelial cells, demonstrating an additive effect (Fig. 4, *A* and *B*). Given the observed discrepancy between transcript and protein levels in LPS-treated FABP3-deficient endothelial cells, we measured the expression level of ICAM1 and VCAM1 in rhFABP3 and LPS–treated endothelial cells. We observed an expected result, where VCAM-1 and ICAM-1 protein were increased in rhFABP3 and LPS–treated endothelial cells in comparison to LPS only–treated endothelial cells (Fig. 4*C*). Next, to assess the effect of rhFAB3 exposure on endothelial cell function *in vivo*, we measured acetylcholine-induced relaxations using myography with isolated aortas from wildtype mice (41). There appears to be a small effect of increasing relaxation (<10%) of phenylephrine-contracted aortas by acetylcholine in the rhFABP3-treatment group *versus* controls; however, the difference was not significant (*p* = 0.5878) (Fig. 4*D*). In order to confirm whether LPS-induced FABP3 expression in endothelial cells *in vitro* also occurs *in vivo*, we treated wildtype mice with 4 mg/kg (42) for 4 h, as we have previously observed that 4 h of LPS treatment is sufficient to induce circulatory cytokines (43) and measured circulatory FABP3 level in mouse plasma. Our data showed significantly increased circulatory FABP3 levels in LPS-treated *versus* vehicle-treated mice (Fig. 4*E*). Taken together, these data indicate that FABP3 exposure exacerbates LPS-induced inflammation *in vitro* and may cause endothelial dysfunction *in vivo* in endothelial cells.

### Loss of FABP3 protects endothelial cells against LPS-induced endothelial dysfunction by promoting cell survival and pro-angiogenic pathways and by inhibiting inflammatory pathways

**Figure 5:**
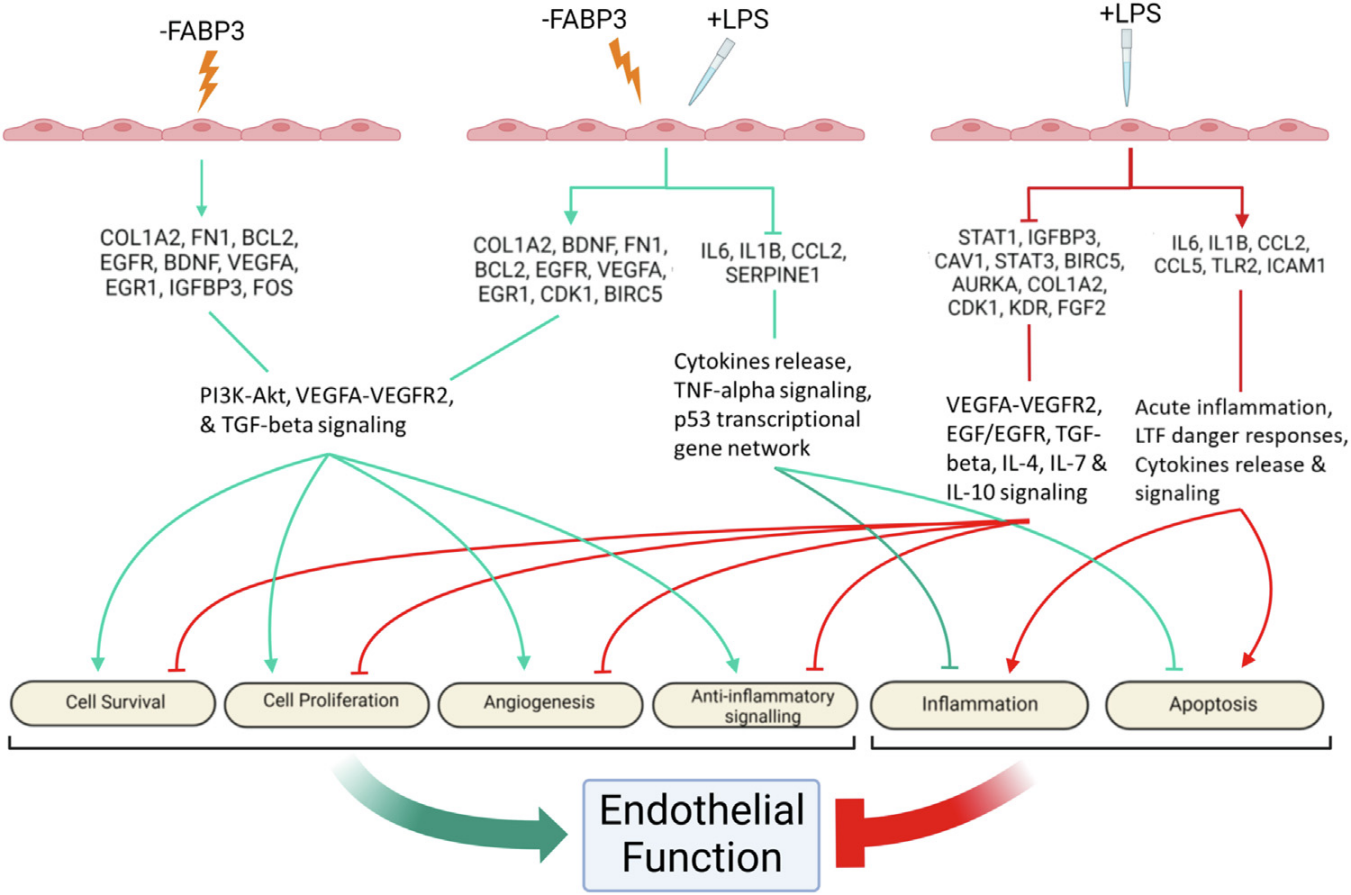
**Endothelial cell-specific loss of FABP3 protects against LPS-induced endothelial dysfunction**. Illustration summarizing the array data on the differentially expressed genes, their regulatory implications, and proposed results in FABP3-deficient endothelial cells. LPS-treated endothelial cells undergo dysfunction, inflammation, and injuries through upregulating and downregulating proinflammatory and prosurvival genes, respectively. Illustration summarizing the array data on the differentially expressed genes, their regulatory implications, and proposed results in endothelial cells under inflammatory stress by LPS. Loss of FABP3 function ameliorates cellular impairment induced by LPS in endothelial cells through upregulation of prosurvival targets and downregulation of inflammatory and senescent factors. Illustration summarizing the array data on the differentially expressed genes, their regulatory implications, and proposed results in LPS stressed with loss of FABP3's function (created with BioRender.com, agreement # KK24HGD240). FABP3, fatty acid–binding protein 3; LPS, lipopolysaccharide.

Given the increased circulatory level of FABP3 in myocardial injury (9) and PAD (18), the obscurity about the role of endothelial FABP3 and the observed complexity about the role of FABP3 in LPS-treated endothelial cells from our data, for clarity, we performed a qPCR array containing 84 endothelial and vascular disease-related genes. Our prime qPCR array data in FABP3-silenced *versus* scrambled control demonstrated a total of 15 upregulated genes (cut-off <2 fold) (Table 1). These genes included pro-angiogenic and prosurvival genes, such as *COL1A2*, *BDNF*, *FN1*, *BCL2*, *EGFR*, *VEGFA*, *EGR1*, *CDK1*, and *BIRC5* (Fig. 5). *PTGS2* was the most upregulated gene identified in the FABP3-silenced endothelial cells. Validation qPCR was performed for five of the upregulated genes to validate the qPCR array data (Table 1). LPS treatment upregulated a total of 10 genes (mainly pro-inflammatory, such as *IL6*, *IL1b*, *CCL2*, *CCL5*, *TLR2*, and *ICAM-1*) and downregulated 18 genes (mainly prosurvival and pro-angiogenic, such as *STAT1, IGFBP3, CAV1, STAT3, BIRC5, AURKA, COL1A2, CDK1, KDR*, and *FGF2*) in comparison to vehicle-treated control (cut-off <2 fold) (Tables 2 and 3 and Fig. 5). *IL1b and MMP7* were the most upregulated and downregulated genes in LPS-treated endothelial cells (Tables 2 and 3). Validation performed for four of the upregulated genes and five of downregulated genes demonstrated a similar trend as the qPCR array (Tables 2 and 3). The prime qPCR array data for LPS *versus* vehicle-treated FABP3-silenced endothelial cells showed a total of 15 upregulated and eight downregulated genes (Tables 4 and 5). Most of the upregulated genes in LPS-treated FABP3-silenced endothelial cells were prosurvival and pro-angiogenic, and the most downregulated genes were proinflammatory in nature (Tables 4 and 5 and Fig. 5). Overall, our PCR array data indicated that loss of FABP3 promotes endothelial cell function and survival and protects against LPS-induced toxicity by promoting pro-angiogenic and prosurvival pathways and by inhibiting inflammation.

**Table 1:** Top upregulated DE mRNAs in HUVECs transfected with siFABP3 *versus* scrambled-controls

qPCR array data				Validation data	
Nr	Gene symbol	Fold change	*p value*	Fold change	*p value*
1	*PTGS2*	5.93	0.001058	3.75 ± 0.61	5.42E-06
2	*COL1A2*	4.04	0.000606	5.68 ± 1.79	3.21E-05
3	*PLAU*	4.01	0.000419	2.00 ± 0.18	0.010158
4	*BDNF*	3.48	0.000012	3.09 ± 0.37	5.01E-08
5	*BCL2*	3.12	0.000104		
6	*CCL5*	2.99	0.03108	2.37 ± 0.32	0.000321
7	*EGR1*	2.93	0.001455		
8	*TLR2*	2.91	0.0044		
9	*EGFR*	2.71	0.003101		
10	*FN1*	2.54	0.005133		
11	*TOP2A*	2.4	0.001877		
12	*RRM2*	2.35	0.005011		
13	*IGFBP3*	2.23	0.010228		
14	*FOS*	2.13	0.025431		
15	*VEGFA*	2.01	0.012069	−	−

Abbreviations: *PTGS2*, prostaglandin-endoperoxide synthase 2; *COL1A2*, collagen type I alpha 2 chain; *PLAU*, plasminogen activator, urokinase; *BDNF*, brain-derived neurotrophic factor; *BCL2*, BCL2 apoptosis regulator; *CCL5*, C-C motif chemokine ligand 5; *EGR1*, early growth response 1; *TLR2*, toll-like receptor 2; *EGFR*, epidermal growth factor receptor; *FN1*, fibronectin 1; *TOP2A*, DNA topoisomerase II alpha; *RRM2*, ribonucleotide reductase regulatory subunit M2; *IGFBP3*, insulin-like growth factor binding protein 3; *FOS*, Fos proto-oncogene, AP-1 transcription factor subunit; *VEGFA*, vascular endothelial growth factor A; DE, differentially expressed.

**Table 2:** Top upregulated DE mRNAs in HUVECs treated with LPS *versus* Vehicle

qPCR array data				Validation data	
Nr	Gene symbol	Fold change	*p value*	Fold change	*p value*
1	*IL1b*	20.01	0.001069	15.1 ± 0.21	0.00007
2	*IL8*	14.87	0.000030	41.1 ± 20.12	0.00193
3	*CCL5*	5.38	0.008772	8.44 ± 1.56	8.42E-05
4	*SOD2*	3.58	0.003152		
5	*ICAM1*	2.82	0.001696	3.47 ± 0.32	0.00021
6	*TLR2*	2.31	0.020022		
7	*CCL2*	2.18	0.035624		
8	*PTGS2*	1.97	0.030868		
9	*IL6*	1.89	0.002871		
10	*PLAU*	1.59	0.039647		

Abbreviations: *IL1b*, interleukin 1 beta; *IL8*, C-X-C motif chemokine ligand 8; *CCL5*, C-C motif chemokine ligand 5; *SOD2*, superoxide dismutase 2; *ICAM1*, intercellular adhesion molecule 1; *TLR2*, toll-like receptor 2; *CCL2*, C-C motif chemokine ligand 2; *PTGS2*, prostaglandin-endoperoxide synthase 2; *IL6*, interleukin 6; *PLAU*, plasminogen activator, urokinase.

**Table 3:** Top downregulated DE mRNAs in HUVECs treated with LPS *versus* vehicle

qPCR array data				Validation data	
Nr	Gene symbol	Fold change	*p value*	Fold change	*p value*
1	*MMP7*	−4.79	0.012612	0.678 ± 0.14	0.091
2	*RRM2*	−4.77	0.000825		
3	*TOP2A*	−4.26	0.000580		
4	*FGF2*	−3.79	0.000975	0.469 ± 0.09	0.0051
5	*CDK1*	−3.68	0.000090	0.661 ± 0.14	0.0013
6	*IL18*	−3.67	0.019809		
7	*BIRC5*	−3.66	0.000739		
8	*COL1A2*	−3.40	0.001588	0.404 ± 0.01	0.0155
9	*TACC3*	−2.92	0.007174		
10	*IGFBP3*	−2.85	0.028628	0.768 ± 0.08	0.068
11	*AURKA*	−2.82	0.019969		
12	*ABCB1*	−2.77	0.000632		
13	*KDR*	−2.45	0.018919		
14	*TCF7L2*	−2.25	0.024388		
15	*RB1*	−2.19	0.006922		
16	*CAV1*	−2.13	0.000385		
17	*STAT1*	−2.13	0.011240		
18	*STAT3*	−2.13	0.032995		

Abbreviations: *MMP7*, matrix metallopeptidase 7; *RRM2*, ribonucleotide reductase regulatory subunit M2; *TOP2A*, DNA topoisomerase II alpha; *FGF2*, fibroblast growth factor 2; *CDK1*, cyclin dependent kinase 1; *IL18*, interleukin 18; *BIRC5*, baculoviral IAP repeat containing 5; *COL1A2*, collagen type I alpha 2 chain; *TACC3*, transforming acidic coiled-coil containing protein 3; *IGFBP3*, insulin-like growth factor binding protein 3; *AURKA*, aurora kinase A; *ABCB1*, ATP binding cassette subfamily B member 1; *KDR*, kinase insert domain receptor; *TCF7L2*, transcription factor 7 like 2; *RB1*, RB transcriptional corepressor 1; *CAV1*, caveolin 1; *STAT1*, signal transducer and activator of transcription 1; *STAT3*, signal transducer and activator of transcription 3.

**Table 4:** Top upregulated DE mRNAs in LPS-treated HUVECs transfected with scrambled-controls *versus* siFABP3

siFABP3- *versus* scramble-transfected and LPS-treated HUVECs				siFABP3- *versus* scramble-transfected HUVECs		LPS- *versus* vehicle-treated HUVECs	
Nr	Gene symbol	Fold change	*p value*	Fold change	*p value*	Fold change	*p value*
1	*COL1A2*	5.46	0.000518	4.04	0.000606	−3.40	0.001588
2	*BDNF*	4.48	0.002061	3.48	0.000012	−1.28	0.045815
3	*RRM2*	3.71	0.000630	2.35	0.005011	−4.77	0.000825
4	*PTGS2*	3.61	0.001994	5.93	0.001058	1.97	0.030868
5	*CCL5*	3.53	0.016385	2.99	0.031080	5.38	0.008772
6	*TOP2A*	3.29	0.004118	2.40	0.001877	−4.26	0.000580
7	*BIRC5*	3.25	0.004793	1.94	0.001717	−3.66	0.000739
8	*BCL2*	3.16	0.006861	3.12	0.000104	−1.27	0.200420
9	*CXCL10*	2.92	0.046103	1.30	0.621431	−1.29	0.685949
10	*EGR1*	2.78	0.008167	2.93	0.001455	−1.30	0.123815
11	*FN1*	2.35	0.013405	2.54	0.005133	−1.83	0.022166
12	*CDK1*	2.33	0.011819	1.42	0.011762	−3.68	0.000090
13	*EGFR*	2.10	0.012967	2.71	0.003101	−1.01	0.939513
14	*TACC3*	2.02	0.019845	1.31	0.184808	−2.92	0.007174
15	*VEGFA*	1.49	0.039754	2.01	0.012069	−1.14	0.488615

Abbreviations: *COL1A2*, collagen type I alpha 2 chain; *BDNF*, brain derived neurotrophic factor; *RRM2*, ribonucleotide reductase regulatory subunit M2; *PTGS2*, prostaglandin-endoperoxide synthase 2; *CCL5*, C-C motif chemokine ligand 5; *TOP2A*, DNA topoisomerase II alpha; *BIRC5*, baculoviral IAP repeat containing 5; *BCL2*, BCL2 apoptosis regulator; *CXCL10*, C-X-C motif chemokine ligand 10; *EGR1*, early growth response 1; *FN1*, fibronectin 1; *CDK1*, cyclin dependent kinase 1; *EGFR*, epidermal growth factor receptor; *TACC3*, transforming acidic coiled-coil containing protein 3; *VEGFA*, vascular endothelial growth factor A.

**Table 5:** Top downregulated DE mRNAs in LPS-treated HUVECs transfected with scrambled-controls *versus* siFABP3

siFABP3- *versus* scramble-transfected and LPS-treated HUVECs				siFABP3- *versus* scramble-transfected HUVECs		LPS- *versus* vehicle-treated HUVECs	
Nr	Gene symbol	Fold change	*p value*	Fold change	*p value*	Fold change	*p value*
1	*MMP1*	−2.21	0.006971	−1.67	0.001441	−1.29	0.023254
2	*CCL2*	−2.01	0.028389	−1.23	0.461531	2.18	0.035624
3	*SERPINE1*	−1.82	0.029037	1.18	0.321398	1.52	0.068618
4	*SOD2*	−1.68	0.045407	−1.22	0.319038	3.58	0.003152
5	*CTBP2*	−1.63	0.047921	−1.25	0.436409	−1.85	0.072752
6	*IL1B*	−2.55	0.054269	1.78	0.208236	20.01	0.001069
7	*IL8*	−1.59	0.062081	1.09	0.388103	14.87	0.000030
8	*IL6*	−1.47	0.058008	1.60	0.005547	1.89	0.002871

Abbreviations: *MMP1*, matrix metallopeptidase 1; *CCL2*, C-C motif chemokine ligand 2; *SERPINE1*, serpin family E member 1; *SOD2*, superoxide dismutase 2; *CTBP2*, C-terminal binding protein 2; *IL1B*, interleukin 1 beta; *IL8*, C-X-C motif chemokine ligand 8; *IL6*, interleukin 6.

## Discussion

The FABPs are cytosolic lipid chaperones abundantly expressed in active lipid-metabolizing tissues, such as the heart and liver, or cell types specialized in lipid storage, trafficking and signaling, such as adipocytes and macrophages (44). The FABP family consists of nine members (FABP 1–9), each with unique tissue-expression patterns (45), although lipid-metabolizing tissues or cells can be found with more than one isoform (46). The degree of FABP-expression in a tissue or cell-type may reflect their lipid-metabolizing capacity, which can be modulated by changes in lipids bioavailability (47). All FABPs are generally known to reversibly interact and escort hydrophobic ligands with various affinities to sites of lipid metabolism or signaling (*e.g.*, lipid droplets, plasma membrane, mitochondria, etc.) (48). However, 20 to 70% sequence homology exists among the nine members (45), and the unique functional features of each member remain poorly understood (49). The FABPs expressed in adipocytes and macrophages have been associated with metabolic and inflammatory regulation (44).

Endothelial cells are known to metabolize fatty acids for energy through mitochondrial oxidation processes (50). Parenchymal absorption of circulating lipids is mediated by endothelial cells (51). Moreover, fatty acids in endothelial cells also have signaling roles impacting cell differentiation, endothelial function, and dysfunction in diseases, although the underlying mechanisms remain largely unclear outside the metabolic diseases. A single recent study has identified FABP3 in human coronary artery endothelial cells and suggested their interaction with the PPARγ through binding fatty acids in regulating transcriptional activities (5). PPARγ is a central component in the inflammatory response mounted by endothelial cells. In addition, the PPAR family of nuclear receptors/transcription factors are expressed in endothelial cells to mediate endothelial function (52). In this notion, we planned to evaluate endothelial FABP3 and investigate its connection to endothelial function. To induce inflammation and endothelial dysfunction, we treated cells with LPS, an *in vitro* model, to study inflammation (26). We used the standard *in vitro* endothelial cell model, HUVECs (53, 54, 55, 56), and confirmed basal FABP3 expression, which was upregulated upon LPS treatment, suggesting a regulatory role of FABP3 in the endothelial response to LPS (Fig. 1, *A*–*C*). LPS binding to endothelial cells elicits endothelial activation, which encompasses the upregulation of inflammatory cytokines and adhesion molecules and the modulation of several critical pathways, including NF-κB, mitogen-activated protein kinase, and PI3K/AKT pathways (57, 58, 59, 60).

Aspects of endothelial function include angiogenesis, migration, proliferation, nitric oxide (NO) production and mounting the inflammatory responses (53, 54, 55, 56). In our tube-formation and migratory assessment, FABP3-silenced HUVECs demonstrated better tube-forming potential, but the migratory potential was reduced relative to scramble controls in both following vehicle or LPS treatment (Fig. 1, *F*–*J*). Although how FABP3 is oppositely influencing the two functional aspects remains inconclusive, our data strongly suggest a consequential role of endothelial FABP3 in angiogenesis and endothelial migration. Endothelial inflammatory activation is marked by an increased migratory response (61). Loss of FABP3 appeared to reduce cell migration at baseline and after LPS treatment, suggesting an independent effect of loss of FABP3 on endothelial cell migration (Fig. 1, *I* and *J*). Endothelial NO synthase is a key regulator of endothelial functions by its influence on NO production, which is essentially involved in oxidative homeostasis and, thereby, influencing many aspects of endothelial function (62). In endothelial cells, AKT is an upstream regulator of eNOS (34). Assessment of these two key regulators of endothelial function revealed an increased eNOS expression in FABP3-silenced HUVECs, and the restoration of eNOS expression in FABP3-deficient endothelial cells following LPS treatment (Fig. 2, *A*–*C*). AKT’s activity, measured by the levels of its phosphorylated and total AKT expression ratio, appeared to be upregulated in both FABP3-silenced endothelial cells and FABP3-silenced endothelial cells treated with LPS (Fig. 2, *D*, *E*, and *H*). Data from both eNOS and AKT assessments suggest their activities are upregulated by the loss of FABP3 in endothelial cells, thereby improving endothelial function. Moreover, LPS has previously been reported to inhibit AKT in endothelial cells (36), but we for the first time show that LPS also significantly inhibits total AKT expression, which was salvaged in endothelial cells with loss of FABP3’s function (Fig. 2, *D*, *E*, and *G*). Likewise, our proliferative and survival assessments indicated improved endothelial proliferation and survival in FABP3-silenced LPS-treated endothelial cells (Fig. 1*K*). P21 is a cyclin-dependent kinase that inhibits the cell cycle and thereby proliferation in endothelial cells (63). LPS is known to promote p21 expression and inhibit cell proliferation (38). In line, we also observed increased p21 expression and reduced proliferation in LPS-treated endothelial cells (Fig. 2, *I*–*K*). However, the loss of FABP3 downregulated p21 both under vehicle and LPS treatment, implying enhanced proliferation and unmasking the effect of LPS (Fig. 2, *I*–*K*). Lastly, our Western blotting data for cleaved-CASPASE3 showed induction of apoptosis in LPS-treated cells (Fig. 1*L*) as previously reported (64); however, the LPS-induced apoptosis was prevented by loss of FABP3 in endothelial cells (Fig. 1*L*). Overall, it appears that in this scenario, both reduced p21 expression and increased survival contribute to the restoration of endothelial cell proliferation in FABP3-defiecient LPS-treated endothelial cells.

In the inflammatory response, activated endothelial cells express adhesion molecules, such as ICAM-1, VCAM-1, and E-SELECTIN, that function primarily to recruit circulatory leukocytes and mediate their transendothelial migration toward the site of acting antigen (65). Activated endothelial cells also secrete the chemokines, such as MCP-1, and the interleukins (*e.g.*, IL1b, IL6, etc.), which mediate the chemotaxis of neutrophils and amplify the inflammatory response, respectively (61). These inflammatory molecules were evaluated in our assessments of endothelial function. LPS is known to induce the expression of endothelial cells inflammatory markers, such as ICAM-1, VCAM-1, and E-SELECTIN (66). Accordingly, we also observed a significant upregulation of these markers in LPS-treated *versus* vehicle-treated endothelial cells (Fig. 3, *A*–*G*). To our surprise, loss of FABP3 significantly reduced LPS-induced *ICAM-1* and *E--SELECTIN* expression; however, interestingly, opposite to *VCAM-1* transcript expression, the VCAM-1 protein expression was significantly exacerbated in LPS-treated FABP3-silenced *versus* LPS-treated control endothelial cells (Fig. 3, *D*–*F*). The observed increase in VCAM-1 protein might be due to a higher protein’s activity and stability in endothelial activation as previously reported (67); however, this remains to be explained. LPS-induced expression of *MCP-1, IL1b, and IL6* were also reduced in FABP3-deficient endothelial cells following LPS treatment (Fig. 3, *H*–*J*).

Establishing the gain-of FABP3’s function through exogenous treatment with rhFABP3 revealed a reverse trend for ICAM-1 and VCAM-1; rhFABP3 exacerbated LPS-induced upregulation of ICAM-1 and VCAM-1 in endothelial cells, reinforcing the inflammatory role of FABP3 (Fig. 4, *A*–*C*). ICAM-1, E-SELECTIN, and VCAM-1 in an activated endothelium all function in leukocyte–endothelial adhesion *via* interaction with leukocytes' LFA-1 (68), PSGL1 (69), and ITGA4/ITGB1 complexes (70), respectively, that are present on leukocytes. Of the three, ICAM-1 is notably also expressed in leukocytes, an active source of fatty acids signaling (71); such interaction may imply a role related to FABP3 in leukocyte–endothelial interaction in an activated endothelium. E-SELECTIN and VCAM-1 are more specific to endothelial cells, and both are notable for their additional roles in angiogenesis (72). Most interestingly, compared to E-SELECTIN and ICAM-1 in endothelial cells, which are localized primarily on the cell membrane, VCAM-1 is expressed both intracellularly in addition to the cell surface (73, 74, 75). This and the diverse regulatory implications of cellular fatty acids (76) may attribute to the complicated behavior of VCAM-1 in our siFABP3-transfected endothelial cells under LPS-induced inflammation. Lastly, the elevation of all three pro-inflammatory markers is associated with cardiovascular and atherosclerotic risk (77). Overall, our data strongly indicate an essential regulatory anti-inflammatory role of FABP3 in endothelial cells. To extend these findings and start to explore the acute effect of exogenous rhFABP3 on vasoreactivity, we performed a myography experiment with isolated aortas from wildtype mice. We treated these aortas with either vehicle or rhFABP3 and measured acetylcholine-induced relaxations. These relaxations did not differ significantly between the controls and the treatment group using 45 ng/ml rhFABP3 (Fig. 4*D*). Although the human and mouse forms of FABP3 are highly conserved, we argue that a larger sample sized study using mouse FABP3 with time-course studies, both sexes, and other blood vessel types, warrants attention as we did observe a small effect of increasing relaxation (<10%). In a clinical scenario, FABP3 released into the circulation following ischemia may help by a vasodilatory effect to increase the blood flow to the impacted tissues. In this scenario, an increase in acute circulatory FABP3 may be beneficial, for example, after acute myocardial infarction; however, a chronic presence of circulatory FABP3 in PAD patients may be beneficial for similar reasons but is countered by the detrimental additive effect to increase the severity of PAD. Accordingly, in PAD, inhibiting FABP3 might prove to be beneficial. LPS is used in an *in vitro* model to study inflammation and in an *in vivo* model to study sepsis (78). To evaluate the relevance of our LPS-associated *in vitro* data in an animal model of sepsis, we treated wildtype mice with LPS and measured circulatory FABP3. Not only we observed baseline circulatory FABP3 but also a significant increase of FABP3 levels in response to LPS stimulation *in vivo* (Fig. 4*E*). The source of LPS-induced FABP3 in mouse plasma is still unknown, but if true in humans, then FABP3 might also provide a biomarker for the severity of sepsis in humans, which warrants future investigations. FABP3 as a biomarker is of particular interest as we were also able to find an association between urinary FABP3 and PAD (79).

**Figure 6:**
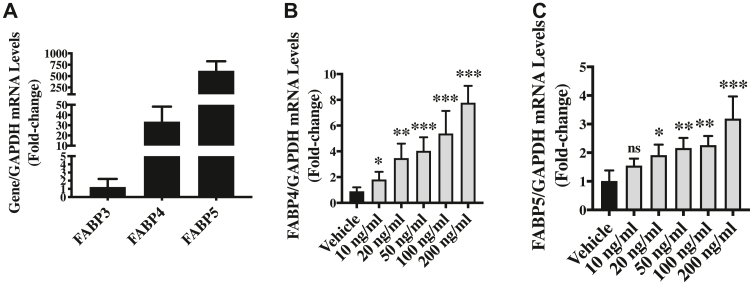
**Relative expression of FABP3, FABP4, and FABP5 in cultured endothelial cells**. *A*, HUVECs were cultured and following 70 to 80% confluency, RNA was extracted to perform qPCR for FABP3, FABP4, and FABP5. *B* and *C*, cultured HUVECs were treated with 100 ng/ml of LPS, and RNA was extracted 24 h posttreatment to perform qPCR for FABP3, FABP4, and FABP5. N = 3 in triplicates. Difference between the means of groups were calculated using ANOVA with Tukey’s multiple comparison test. ∗*p* < 0.05, ∗∗*p* < 0.01, ∗∗∗*p* < 0.001 *versus* vehicle. FABP3, fatty acid–binding protein 3; HUVECs, human umbilical vein endothelial cells; LPS, lipopolysaccharide.

Given that FABP4 and FABP5 are the known predominant FABPs in endothelial cells (1, 4), we assessed the relative expression of *FABP3, FABP4, and FABP5*. As expected, out of these three FABPs, *FABP5* was the most, and *FABP3* was the least expressed FABP in endothelial cells (Fig. 6*A*). These data show that FABP3 is basally expressed at a low level; however, *FABP3* is upregulated in stress conditions such as LPS-treatment in endothelial cells. Next, we also tested the effect of LPS on *FABP4* and *FABP5* and observed similar upregulation of these genes similar to *FABP3* (Fig. 6, *B* and *C*). FABP4 and FABP5 are known to be co-expressed and to play overlapping as well as nonredundant roles (1, 4). A similar pattern observed for LPS-induced upregulation of *FABP3, FABP4, and FABP5* in endothelial cells indicates that these molecules may be co-expressed; however, distinct effects of loss-of and gain-of FABP3 in endothelial cells warrant similar investigations following the loss-of and gain-of FABP4 and FABP5 in endothelial cells under LPS treatment or inflammation.

To understand the complexity of FABP3 action and to expand our endothelial function assessment, the regulatory roles of endothelial FABP3 were conducted using a prime qPCR array to evaluate endothelial cell-specific genes known to play roles in vascular disease. Among the upregulated genes in siFABP3-transfected endothelial cells, *COL1A2* encodes for collagen type I, which composes the extracellular matrix and surrounding connective tissues. The expression of *COL1A2*, as well as that of the fibronectin-encoding *FN1* gene, are featured in the focal adhesion processes of endothelial cells, which promote endothelial cells’ integrity, growth, and survival through the TGF-beta and PI3K-AKT signaling pathways (80, 81). *EGFR’s* gene product, which is the receptor for the epidermal growth factors, is also activated in endothelial focal adhesion and promotes cell growth through the PI3K-AKT signaling pathway (82). *IGFBP3*, which encodes for a component of the complex-carrier of the insulin-like growth factors, stimulates endothelial cells’ proliferation through EGFR (83, 84). *VEGFA* is an inducer of endothelial cell growth required for angiogenesis and vasculogenesis, as well as general endothelial function and the maintenance of vasculature’s integrity (85). As we observed upregulated *EGR1, VEGFa* also promotes EGR1 (86), which has been linked to activated PDGF-A that regulates endothelial function in endothelial cells (87). *FOS’s* gene product, which composes the transcription factor complex AP-1, promotes endothelial cell growth (88). *BDNF*, whose gene product is essential in the survival and differentiation of neurons (89), regulates vessel’s integrity and promotes angiogenesis in endothelial cells (90). Lastly, *BCL2* is the prosurvival factor in apoptosis, which functions to inhibit caspases activity, thereby promoting cell survival (91). Overall, the upregulation of these genes in HUVECs with compromised FABP3 indicates a positive regulation of endothelial cells’ growth, function, and survival from the loss-of FABP3’s function (Table 1 and Fig. 5).

Under LPS treatment, endothelial cells respond negatively with impaired function and survival and a state of inflammation (Tables 2 and 3 and Fig. 5). As expected, in LPS-treated HUVECs, genes encoding for the pro-inflammatory cytokines *(IL6, IL1B)* and chemokines *(CCL2, CCL5)* were upregulated, as well as *TLR2*, which encodes for the receptor responding against foreign agents (25), and the leucocyte-adhesive inflammatory marker *ICAM-1* (92). Likewise, among the downregulated genes were *IGFBP3 and COL1A2*, indicating a downregulation in endothelial proliferation and survival in response to LPS. This notion is further supported by the downregulated *AURKA, FGF2, and CDK1*, whose gene products are key regulators of cellular proliferation (93, 94, 95). The gene products of *STAT1 and STAT3* are transcription factors of the STAT protein family known to be regulated by the interferons and EGFs (96, 97); their roles have also been implied in VEGFa and EGFR signaling (98, 99), as well as the production of the anti-inflammatory cytokines IL-4, IL-7, and IL-10 (100). Downregulated *STAT1 and STAT3* in LPS-treated endothelial cells, therefore, indicated compromised cell growth and a state of pro-inflammation. Impaired endothelial function is further suggested by the downregulation of *CAV1*, a mediator of cellular transcytosis essential for many cell-signaling pathways (101), and *KDR* that encodes for VEGFR, the receptor for VEGFa (102). LPS-mediated apoptosis is also indicated by downregulated *BIRC5*, whose gene product, Survivin, is a member of the inhibitor of apoptosis (IAP) protein family (103).

From the differentially expressed genes in HUVECs with both siFABP3 and LPS treatments, we observed a remarkable ameliorating effect by the loss of FABP3’s function (Tables 4 and 5 and Fig. 5). Salvaged endothelial integrity and survival were indicated by the upregulation of *COL1A2 & FN1 and BCl2 & BIRC5, respectively. Upregulated EGFR and CDK1 further suggested increased cell proliferation, and upregulated BDNF, EGR1, and VEGFA indicated a promotion of endothelial functions. On the other hand, CCL2 as well as IL6 and IL1B were downregulated, indicating reduced inflammation. Interestingly, SERPINE1, whose gene product is an inhibitor of fibrinolysis* (104), *was downregulated*. Activated SERPINE 1 also promotes cellular senescence downstream of the p53 regulatory network (105). Reduced activity of SERPINE1, therefore, suggests that loss of FABP3’s function prompts endothelial cells away from a state of senescence and improves clot breakage, providing benefits against cardiovascular risk in aging and dysregulated clot formation in atherosclerosis (106).

In summary, our data demonstrate that FABP3 is expressed in endothelial cells and that loss of endothelial FABP3 inhibits LPS-induced endothelial dysfunction by modulating cell survival and inflammatory and angiogenic signaling pathways. We also observed exacerbation of LPS-induced inflammation in endothelial cells. We were able to provide a global view of the pathways associated with FABP3; however, these findings warrant further detailed investigations. We observed a rather low expression of FABP3 in endothelium and an increased level of circulating FABP3 in LPS-treated mice; however, it remains to be seen whether the endothelium is a significant source of FABP3 in LPS-treated mice and in PAD patients. To this aim, we are generating endothelial cell-specific FABP3 knockout (FABP3^endo^) mice. We will measure circulating FABP3 following LPS treatment to FABP3^endo^ and wildtype mice. Circulating FABP3 will also be measured following crossing FABP3^endo^ mice with ApoE^null^ mice (FABP3^endo^:ApoE^null^) and feeding them high-fat diet to induce atherosclerosis. A decreased level of circulating FABP3 in FABP3^endo^ following LPS-treatment or in FABP3^endo^:ApoE^null^ mice following high-fat diet treatment will confirm endothelium as a significant source of FABP3 *in vivo*. As of current, our data indicate that an increase in circulating FABP3 may be detrimental to endothelial function, and therefore, therapies aimed at inhibiting FABP3 may improve endothelial function in diseased states, particularly in the cases with chronic elevation of FABP3, such as PAD.

## Experimental procedures

### Animals

Wildtype C57BL/6J (Charles River Laboratories) were used in accordance with the Guide to Care and Use of Animals of the Canadian Council of Animal Care (CCAC). The use of animals was approved by the Animal Care Committee at Western University, Canada.

### Cell culture, FABP3 silencing, and LPS treatment

HUVECs (Lonza # CC-2519, pooled, passage # 4–7), a standard model to study endothelial cells function *in vitro* (28, 53, 54, 55, 56), were grown in endothelial cell complete growth medium-2 (EGM-2 Bulletkit; Lonza). HUVECs were transfected with either siFABP3 (optimized to 5 nM, sense strand: 5′-GCUAAUUGAUGGAAAACUCTT -3′ and antisense strand: 5′-GAGUUUUCCAUCAAUUAGCTC-3′) or scrambled control (Ambion Silencer Select Pre-Designed siRNA) using Lipofectamine RNAi-max (Invitrogen) and OptiMEM (Gibco). Following 24 h of transfection, HUVECs were treated with either LPS (Sigma-Aldrich) or diluent (PBS) in MCDB-131 low-serum media (+1% FBS) for different time points. HUVECs were starved overnight in the MCDB-131 low-serum media before treatment.

### RNA extraction, cDNA synthesis, and quantitative real-time PCR

Following transfection and treatment, total RNAs were extracted and quantified using the Trizol standard method (Invitrogen) (107). Total RNA was quantified using NanoDrop (Thermo Scientific). Complementary DNAs were synthesized from RNAs using the QuantiTect kit (Qiagen). qPCRs were conducted to evaluate the expression of targeted genes using SYBR (Bio-Rad), primers, and QuantStudio-3 Real-Time PCR system (Applied Biosystems). All protocols were conducted in accordance with the manufacturer’s instructions. The qPCR was performed for *GAPDH, vascular cell adhesion molecule-1 (VCAM-1), intercellular adhesion molecule-1 (ICAM-1), E-SELECTIN* (108), *p21, eNOS* (53), *FABP3 (forward 5′-CATGACCAAGCCTACCACAAT-3′ and reverse 5′-CCCCAACTTAAAGCTGATCTCTG), FABP4* (109), *FABP5* (109), *IL1b (forward 5′-GAAGCTGATGGCCCTAAACA-3′ and reverse 5′- AAGCCCTTGCTGTAGTGGTG-3′), IL6 (forward 5′-AGTGAGGAACAAGCCAGAGC-3′ and reverse 5′-GTCAGGGGTGGTTATTGCAT-3′), MCP1 (forward 5′-GCCTCCAGCATGAAAGTCTC-3′ and reverse 5′-AGGTGACTGGGGCATTGAT-3′),* and *AKT (forward 5’ -TCTATGGCGCTGAGATTGTG-3′ and reverse 5′-CTTAATGTGCCCGTCCTTGT-3′)*.

### Western blot

Cultured HUVECs were collected in RIPA buffer to isolate total proteins (110). Equal amount of proteins from each sample were loaded onto sodium dodecyl sulfate (SDS) polyacrylamide gels, which were then subjected to electrophoresis. Proteins were then transferred onto PVDF membranes (Bio-Rad), and the following antibodies were employed to detect for the proteins of interest [Cell Signaling Technology: ICAM-1 (4915S, dilution 1:1000), VCAM-1 (13662S, dilution 1:1000), eNOS (32027S, dilution 1:1000), phospho (p)-eNOS (Millipore, 07 -428-I, dilution 1:1000), AKT (4691S, dilution 1:1000), p-AKT (4060S, dilution 1:1000), cleaved-CASPASE3 (9664S, dilution 1:1000), p21 (2947S, dilution 1:1000), and GAPDH (5174S, dilution 1:1000)]. Western blot for FABP3 was performed using polyclonal antibody (ThermoFisher, PA5-92386, dilution 1:1000), and wildtype mouse total heart protein was used as a positive control. Western blots were developed using chemiluminescence substrates (Bio-Rad) and the Licor-Odyssey XF Imaging System. Densitometry was performed to measure the band intensities using the Image Studio Lite.

### Cell counting

HUVECs were silenced and seeded at a density of 2 × 10^5^ cells/well in a 6-well plate prior to LPS or diluent control for 24 h. Cells from each well were then harvested and counted under an Automated Cell Counter (CytoSmart) to assess for proliferative/viability capacity.

### Scratch assay

Following reverse transfection, HUVECs were seeded at a density of 2 × 10^5^ cells/well in a 6-well plate and allowed to grow to 70 to 80% confluency. Each well was then administered a consistent straight scratch prior to LPS or negative control. Phase-contrast microscopy using an adapted camera (Optika) was employed to take pictures of cells in each well migrating into the scratch over time to evaluate for migrating capacity as described (111). The experiment was performed in triplicates.

### *In vitro* tube-formation assay

The *In vitro* Angiogenesis Kit (Millipore) was employed to evaluate endothelial angiogenic properties. HUVECs were transfected and seeded at a density of 2 × 10^5^ cells/well in a 6-well plate and allowed to grow to ∼75% confluency. The kit-provided matrix solution was added into designated wells of a 96-well plate. Transfected cells from the previous preparation were then harvested and seeded at an equal density of 1 to 1.5 × 10^4^ cells/well onto the designated wells in EGM-2 supplemented with LPS or vehicle diluent. Phase contrast microscopy was employed (Optika) to obtain pictures of cells under phase contrast in each designated well over time to monitor tube formation, and quantification was performed according to the manufacturer’s instruction.

### Exogenous recombinant FABP3 treatment

HUVECs grown in endothelial cell complete growth medium-2 were exposed to different doses of human-recombinant FABP3 (Cayman Chemical) or their diluent (PBS) in low-serum MCDB-131 media. Following 1 h of exposure, HUVECs were treated with either LPS or PBS for 6 h for RNA extraction to perform qPCR and 24 h for protein extraction to perform Western blotting.

### Enzyme-linked immunosorbent assay and wire myography

HUVECs were cultured and treated with 100 ng/ml of LPS for 24 h following 80% confluency. Later, culture medium was collected, and ELISA for FABP3 was performed using concentrated culture medium and analyzed as instructed by DuoSet ELISA Development System and Ancillary Reagent Kit 2 (R&D Systems, Cat. # DY1678 and DY008). ELISA for circulating FABP3 was performed following 4 h of i.p. injection of LPS (4 mg/kg) or vehicle (PBS) to the wildtype mice (N = 6/group, C57BL/6 12–15 weeks old male—Charles River Laboratories). Blood was collected in heparinized tubes, centrifuged, and the supernatant was collected to isolate plasma. A total of undiluted 100 μl of mouse plasma was used to perform ELISA as instructed by the Mouse FABP3 ELISA Kit (FroggaBio, Cat #MOES01684).

### Isometric tension myography studies of isolated aortas

Wildtype male mice (n = 4, 12–15 weeks of age) were euthanized by overdose inhalation of isoflurane. Descending thoracic aortas were removed from mice and placed in ice-cold Krebs Hepes buffer while cleaned of adherent fat and connective tissues. Krebs Hepes buffer (pH 7.4, 37 °C) was composed of 114 mM NaCl, 4.7 mM KCl, 0.8 mM KH_2_PO4, 1.2 mM MgCl_2_ 6H_2_O, 2.5 mM, CaCl_2_ 2H_2_O, 11.0 mM D-Glucose, 20 mM NaHCO_3_, and 5 mM Hepes hemisodium salt. Krebs buffer was bubbled continuously with 95% O_2_/5% CO_2_ during myograph experiments. In brief, we used DMT 620M myograph chambers with the methods and conditions described in (41) for continuous measuring and recording of isometric tension with mouse aortas. The aorta from each mouse was divided into two groups: control (vehicle, PBS) and treatment (rhFABP3, 45 ng/ml). We tested the viability of aorta preparations (1–3 mm lengths) using 90 mM KCl. Viable tissues contractions were >1 mN. We assessed acetylcholine-induced relaxations of phenylephrine-contracted aortas under isometric tension conditions as we described previously (41). Aortic rings mounted in the DMT620 M chambers were exposed to treatments for 20 min then contracted with phenylephrine (3 μM) and then acetylcholine dose–responses curves constructed.

### Prime qPCR array

RNAs extracted from HUVECs transfected with either siFABP3 or scrambled control and treated with LPS or vehicles were subjected to a prime qPCR array screening a library of vascular disease–related genes (Bio-Rad, Vascular disease, tier 1, H384, cat#10038720). The expression levels of the differentially expressed mRNAs targets were measured and then organized to outline the topmost upregulated or downregulated gene targets. HUVECs were treated with either LPS or diluent in MCDB-131, and RNAs were extracted to perform validation qPCR. Validation of the outlined targets were then conducted by regular qPCR procedure using the primers listed in Table S1 as described in the *RNA Extraction, cDNA Synthesis, and Quantitative Real-Time PCR section*. The targets were also analyzed by gene ontology enrichment using Enrichr software to highlight the biological processes or pathways affected by the differentially expressing genes.

### Data and statistical analysis

Difference between the means of two groups and more than two groups were calculated using the Student’s *t* test and analysis of variance (ANOVA) statistical analysis, respectively. ANOVA significant results were followed by the post-hoc Tukey’s test. Data are presented as mean ± SD unless otherwise indicated. N = number of independent experiments or animals. In myograph experiments, relaxation (%) by acetylcholine was calculated as the reversal of tension induced by the contractile agonist (phenylephrine). Acetylcholine-induced relaxations were analyzed using 2-way ANOVA with Bonferroni post-hoc test for pairwise comparisons. *p* <0.05 was considered significant.

## Data availability

Data will be available upon request.

## Supporting information

This article contains supporting information.

## Conflict of interest

The authors declare no conflict of interest with the contents of this article.
