# Supplementary material for: Loss of fatty acid binding protein 3 ameliorates lipopolysaccharide-induced inflammation and endothelial dysfunction
Source: J Biol Chem. 2023 Jan 19;299(3):102921. doi: 10.1016/j.jbc.2023.102921 (PMC9988587; doi:10.1016/j.jbc.2023.102921)
Supplement: Supplemental table [file mmc1.docx]

| **Targets** | **Forward primer's sequence** | **Reverse primer's sequence** |
| --- | --- | --- |
| ***PTGS2*** | 5’-CTGGCGCTCAGCCATACAG-3’ | 5’-CGCACTTATACTGGTCAAATCCC-3’ |
| ***Col1A2*** | 5’-GGCCCTCAAGGTTTCCAAGG-3’ | 5’-CACCCTGTGGTCCAACAACTC-3’ |
| ***PLAU*** | 5’-GGGAATGGTCACTTTTACCGAG | 5’-GGGCATGGTACGTTTGCTG-3’ |
| ***BDNF*** | 5’-GGCTTGACATCATTGGCTGAC-3’ | 5’-CATTGGGCCGAACTTTCTGGT-3’ |
| ***IL8*** | 5’-ACTGAGAGTGATTGAGAGTGGAC-3’ | 5’-AACCCTCTGCACCCAGTTTTC-3’ |
| ***SPARC*** | 5’-CCCATTGGCGAGTTTGAGAAG-3’ | 5’-CAAGGCCCGATGTAGTCCA-3’ |
| ***MMP7*** | 5’-GAGTGAGCTACAGTGGGAACA-3’ | 5’-CTATGACGCGGGAGTTTAACAT-3’ |
| ***SERPINE*** | 5’-GCACCACAGACGCGATCTT-3’ | 5’-ACCTCTGAAAAGTCCACTTGC-3’ |
| ***TYMS*** | 5’-GGAGTGAAAATCTGGGATGCC-3’ | 5’-ACTGGAAGCCATAAACTGGGC-3’ |
| ***FGF2*** | 5’-AGTGTGTGCTAACCGTTACCT-3’ | 5’-ACTGCCCAGTTCGTTTCAGTG-3’ |
| ***CDK1*** | 5’-GGATGTGCTTATGCAGGATTCC-3’ | 5’-CATGTACTGACCAGGAGGGATAG-3’ |
| ***RB1*** | 5’-TTGGATCACAGCGATACAAACTT-3’ | 5’-AGCGCACGCCAATAAAGACAT-3’ |
| ***KRAS*** | 5’-GGACTGGGGAGGGCTTTCT-3’ | 5’-GCCTGTTTTGTGTCTACTGTTCT-3’ |
| ***VEGFR2*** | 5’-GTGATCGGAAATGACACTGGAG-3’ | 5’-CATGTTGGTCACTAACAGAAGCA-3’ |
| ***IGFBP3*** | 5’-AGAGCACAGATACCCAGAACT-3’ | 5’-GGTGATTCAGTGTGTCTTCCATT-3’ |

**SUPPLEMENTARY TABLE 1**

**Supplementary Table 1.** List of primers used to amplify respective genes (source: https://pga.mgh.harvard.edu/primerbank/).
